# Internet-based Self-Management Support for Patients With Well-Controlled Type 2 Diabetes: A Real-Life Study

**DOI:** 10.2196/resprot.6910

**Published:** 2017-03-23

**Authors:** Huberta E Hart, Inge ETM Geilen, Elke de Leeuw, Guy EHM Rutten, Rimke C Vos

**Affiliations:** ^1^ University Medical Center Utrecht Julius Center for Health Sciences and Primary Care Utrecht Netherlands; ^2^ Leidsche Rijn Julius Health Centers Utrecht Netherlands

**Keywords:** type 2 diabetes mellitus, self-care, telemedicine, mild cognitive impairment

## Abstract

**Background:**

Little attention has been paid to self-management support of patients with well-controlled type 2 diabetes mellitus (T2DM). Most studies evaluated the addition of self-management support to regular diabetes care, but self-management as an alternative for part of regular diabetes care has hardly been studied. In this study, we offered patients with well-controlled T2DM the opportunity to perform the 3 quarterly monitoring sessions at home using an Internet-based self-management program, resulting in online personalized advice.

**Objective:**

The aim of our study was to assess the reach and feasibility of an Internet-based diabetes self-management support program for patients with well-controlled T2DM, addressing both primary care providers’ (PCPs) opinions and patients’ willingness to participate in such a support program.

**Methods:**

PCPs assessed patients’ eligibility for Internet-based self-management, and patients were offered the opportunity to participate. Characteristics of eligible and ineligible patients were compared, as well as those of participants and nonparticipants, also with regard to quality of life, treatment satisfaction, and illness perceptions. Multivariate logistic regression models were performed and odds ratios (ORs) calculated with 95% CIs.

**Results:**

Almost half (128/282, 45.4%) of the patients with well-controlled T2DM were considered ineligible by their PCPs mainly because of cognitive impairment and language barriers (8.2% and 8.9%). Older patients (OR for each year 1.06, 95% CI 1.03-1.09, *P*<.001), non–Western European patients (OR 3.64, 95% CI 1.67-7.92, *P=*.001), and patients with a longer diabetes duration (OR for each year 1.56, 95% CI 1.04-2.34, *P*=.03) were more often regarded as ineligible. Of the 154 patients considered eligible, 57 (37.0%) consented to participate and 30 (10.6%) started the program. Of 57 participants, 45 returned the 3 questionnaires; 21 of 97 nonparticipants returned the questionnaires. Nonparticipants less often thought that their disease would last their entire life (median 8.0 vs 10.0, *P*=.03) and they were more satisfied with their current treatment than participants (DTSQ total score 44.0 vs 40.0, *P*=.05). There was no significant difference in quality of life between the 2 groups.

**Conclusions:**

PCPs considered half of their patients with well-controlled T2DM incapable of Internet-based self-management mainly because of cognitive impairment and language barriers; of the selected patients, about 1 out of 3 was willing to participate. Older patients, non–Western European patients, and patients with a higher BMI were less likely to participate. Predominantly, practical issues (such as Internet problems) hindered implementation of the Internet-based self-management program.

## Introduction

In response to the expanding impact of type 2 diabetes mellitus (T2DM) on health care systems, research has focused on the effectiveness of strategies to improve diabetes self-management. Diabetes self-management support is not restricted to the patient-provider encounter; it needs to be an ongoing process [1]. The type of support can be behavioral, educational, psychosocial, or clinical. Over the last few years, the approach has changed from a didactic one, providing information, to a more empowering type of support, focusing on helping those with diabetes make informed self-management decisions [2,3]. Ideally, empowered patients develop personal goals together with their health care provider and make daily decisions in tuning the management of their disease to circumstances [3]. With diabetes self-management education (DSME), the skills and abilities necessary for diabetes self-care are facilitated in an ongoing fashion [2,4]. We speak of diabetes self-management education and *support* (DSME/S) to underline the importance of ongoing support for individuals with diabetes, particularly to encourage behavioral change, the maintenance of healthy diabetes-related behaviors, and to address psychosocial concerns. Strategies supporting DSME/S are diverse, for example, using telephone follow-up calls or Web-based technologies [1].

Self-management support research mainly focuses on improving self-management of patients with poorly controlled T2DM; less attention has been paid to the support and skills of individuals with well-controlled T2DM. Moreover, research mostly evaluates the *addition* of self-management education or support to regular diabetes care but hardly evaluates the promotion of self-management support as an *alternative* for part of regular diabetes care [5-8]. Patients with well-controlled T2DM, with assumed good self-management skills and behaviors, might benefit from an individualized treatment approach that requires less frequent monitoring by their health care provider. Indeed, glucose levels, blood pressure, and lipid levels in patients with well-controlled T2DM who received 2 checkups per year did not differ from patients who received 4 checkups per year. These results suggest sufficient self-management competence of patients with well-controlled T2DM to maintain adequate cardiometabolic control [9]. However, offering patients the choice of different number of practice visits (2, 3, or 4 times per year) is not yet usual care. Whether just 1 annual checkup at the health care center in combination with adequate self-management support might be sufficient is not known.

Internet-based self-management programs offer new opportunities for patients to practice diabetes self-management at home at a convenient time; they might be less time-consuming for both the patient and the primary care provider (PCP) [5,10]. Nurses estimate the self-care capacities of their patients lower than patients themselves [11]. Practicing self-management is not only related to cardiometabolic control, but also to other aspects of diabetes as a chronic condition, such as health-related quality of life, treatment satisfaction, and illness perceptions [12]. Because ethnic differences, sex, and comorbidities can influence quality of life and illness perceptions, they might also determine patients’ diabetes self-management [13]. Health care providers should therefore consider those aspects when providing self-management support [14].

We aimed to determine the reach and feasibility of an Internet-based diabetes self-management support program for patients with well-controlled T2DM, addressing both PCPs’ opinions and patients’ willingness to participate in Internet-based self-management and investigating the role of treatment satisfaction, health-related quality of life, and illness perceptions in this respect.

## Methods

### Study Design and Participants

The study was conducted among 36 PCPs (26 general practitioners and 10 practice nurses) in 4 primary care centers of the Leidsche Rijn Julius Health Centers in Utrecht, the Netherlands, delivering care to 890 T2DM patients. In a previous study, patients with well-controlled T2DM were selected on the basis of their individualized treatment targets for hemoglobin A_1c_ (HbA_1c_), systolic blood pressure (SBP), and low-density lipoprotein (LDL) cholesterol as defined in the Dutch guidelines for T2DM and cardiovascular risk management [15]. According to the Dutch individualized approach for HbA_1c_, individuals aged <70 years and those aged ≥70 years with lifestyle advice only or receiving metformin monotherapy should achieve an HbA_1c_ target of ≤53 mmol/mol (≤7%). Those aged ≥70 years who are using more blood glucose–lowering agents than metformin and with a diabetes duration of less than 10 years should achieve an HbA_1c_ level of ≤58 mmol/mol (≤7.5%); those with diabetes duration more than 10 years should achieve an HbA_1c_ level of ≤64 mmol/mol (≤8%) [16]. The individualized target level for SBP depends on age; patients aged <80 years should achieve an SBP of ≤140 mm Hg, and those aged ≥80 years should achieve an SBP of ≤160 mm Hg [16,17]. Only in patients with an indication for primary or secondary prevention of cardiovascular disease, the target level for LDL cholesterol is ≤2.5 mmol/L. To determine whether primary prevention is needed, the Dutch guideline uses the Systematic Coronary Risk Evaluation (SCORE) risk function, based on age, sex, smoking status, SBP, and total cholesterol/high-density lipoprotein cholesterol ratio, to determine the 10-year fatal and nonfatal cardiovascular disease risk [17]. Because of the increased risk of cardiovascular disease among patients with T2DM, 15 years is added to the calendar age of patients with T2DM to determine their 10-year cardiovascular disease risk from the SCORE risk function. Patients with a 10-year risk greater than 20% have an indication for primary prevention and thus a target LDL level of ≤2.5 mmol/L. The same holds for patients with 10%-20% risk and with one or more additional risk factors, that is, poor metabolic control, microalbuminuria, overweight, decreased estimated glomerular filtration rate (eGFR), reduced physical activity, or a positive family history of cardiovascular disease. Secondary prevention is indicated in all patients with macrovascular disease [17]. According to these Dutch guidelines, 282 patients (31.7%) had good cardiometabolic control and were eligible to be included in this study [15].

First, the general practitioners, in collaboration with the practice nurses, were asked to judge the eligibility of their patients with well-controlled T2DM and to motivate them to participate in an Internet-based self-management program (see below) to replace 3 out of the 4 regular diabetes monitoring visits.

Second, the patients eligible for Internet-based self-management were offered the opportunity to participate in the Internet-based self-management support program. They could mark their preference and motivation to participate in the Internet-based self-management support program or to continue their care as usual on a return form. If patients decided to participate, they gave informed consent during the next regular practice visit and were enrolled in the study. They performed their first Internet-based self-monitoring session 3 months after their enrollment.

Third, all eligible patients received 3 validated questionnaires regarding quality of life, treatment satisfaction, and illness perceptions before they started the Internet-based self-management (see below).

All available data of participants were collected and the database was locked 1 year after the enrollment of the first patient.

The study was approved by the Medical Research Ethical Committee of the University Medical Center Utrecht.

### Internet-based Self-Management Program

Individualized treatment goals were set for the patients with well-controlled T2DM in collaboration with their PCP, during the last practice visit before the enrollment into the Internet-based self-management program. The Internet-based self-management support system was explained to the patients as an alternative for 3 out of 4 regular diabetes checkup visits at the primary care center. Every 3 months, patients received an Internet-based reminder to perform the Internet-based quarterly monitoring. If the patient did not perform the monitoring, he or she received a second reminder. The monitoring consisted of two parts. First, patients were asked about their physically and mentally perceived health in the preceding 3 months and more specifically about their body weight, the presence of diabetic ulcers, their feet, and about cardiovascular problems. Also, medication adherence and medication side-effects were registered. Second, the current weight, fasting blood glucose level, and blood pressure were self-measured and filled in, for which purpose patients had to possess or buy blood glucose and blood pressure measuring devices (cost: €20 in total). On the basis of the entered data, patients received advice, for example, “contact your PCP directly/next working day.” Advice was based on predefined cutoff values for blood glucose, blood pressure, and answers on the questions about physically and mentally perceived health and medication adherence and/or medication side-effects.

### Patient Characteristics

Characteristics of all patients with well-controlled T2DM were retrieved from electronic patient records in August 2014 and included age, sex, ethnicity (“Western European” or “non–Western European” based on country of origin of their parents), educational level, duration of diabetes, body mass index (BMI), the presence of microvascular complications and cardiovascular disease, and type of treatment (lifestyle advice only, oral blood glucose–lowering agents, insulin). Educational level was classified as low, middle, or high, according to the Dutch National Public Health Compass [18]. Registered microvascular complications were diabetic nephropathy (eGFR<30 mL/min/1.73m^2^ or presence of macroalbuminuria), retinopathy, or neuropathy (SIMMS classification ≥1). Macrovascular diseases including angina pectoris, myocardial infarction, chronic ischemic heart disease, transient ischemic attack, cerebral infarction, intermittent claudication, or aortic aneurysm were recorded.

### Questionnaires

#### Quality of Life

The EQ-5D consists of 5 dimensions: mobility, self-care, usual activities, pain or discomfort, and anxiety or depression, each with 3 options of choice ranging from 1 (no problems) to 3 (severe problems). The EQ-5D health state utility scores range from −0.33 to +1.00 and were computed using the Dutch tariff as described by Lamers et al [19,20]. A score of 0 is equal to death, whereas 1 indicates full health. Negative values represent a health state worse than death, meaning an extreme low quality of life. The EQ visual analogue scale (EQ VAS) is a scale ranging from 0 to 100, where respondents can rate their overall health state. A value of 0 indicates the worst imaginable health state, whereas 100 indicates the best imaginable health state [21].

#### Diabetes Treatment Satisfaction

The Diabetes Treatment Satisfaction Questionnaire (DTSQ) includes 8 items: overall treatment satisfaction, frequency of hyperglycemia and hypoglycemia, treatment convenience and flexibility, satisfaction with understanding of diabetes, and willingness to continue the present treatment and to recommend it to others. All items can be scored ranging from 0 (eg, very dissatisfied) to 6 (eg, very satisfied), with a total score that ranges from 0 to 48. To calculate this total score, the Likert scales used for measuring the frequency of hypoglycemia and hyperglycemia were reversed [22].

#### Illness Perceptions

The Brief Illness Perception Questionnaire (BIPQ) consists of 9 questions. The first 8 items are scored on an 11-point Likert scale, ranging from 0 to 10, with a different meaning for each question: consequences (the impact of the disease on daily life), timeline (duration of the disease), personal control, treatment control, identity (symptoms experienced), concern, understanding, and emotional response. The ninth question is open-ended and consists of mentioning the 3 most important causes of the disease according to the patient [23,24].

### Statistical Analysis

To determine differences between eligible and ineligible patients and between participants and nonparticipants, descriptive statistics were performed. Categorical variables are reported as counts and percentages, continuous variables as means with SD or medians with interquartile range (IQR) for nonnormally distributed variables. The chi-square test was used to assess differences between groups for categorical variables, the unpaired *t* test for normally distributed continuous variables (age, EQ VAS), and the Mann-Whitney *U* test for nonnormally distributed continuous variables. To analyze the BIPQ items, Mood’s median test and the Mann-Whitney *U* test were used. To determine which variables were independently associated with eligibility for Internet-based self-management according to the PCPs, multivariate regression analyses were used with eligibility as the dependent variable, adjusted for clustering at practice level. Included determinants were age, sex, diabetes duration (square root transformed), microvascular complications, cardiovascular disease, using insulin, “lifestyle advice as only treatment,” and BMI. Because data on ethnicity were missing for 12% of the patients, ethnicity was included in a second model to analyze its association with eligibility of patients for Internet-based self-management.

To determine which variables were independently associated with participation in the Internet-based self-management support program, multivariate regression analysis was performed, with participation as the dependent variable, adjusted for clustering. On the basis of the results of the first logistic regression analysis and clinical relevance, the following determinants were selected: age, diabetes duration, ethnicity (no missing data), microvascular disease, cardiovascular disease, and BMI.

Results of the logistic regression models are presented as odds ratios (ORs) with 95% CIs and *P* values. A *P* value of <.05 was considered statistically significant. IBM SPSS Statistics version 22 (IBM Corporation) was used.

## Results

### Health Care Providers

All PCPs, 26 general practitioners and 10 practice nurses, participated. Their mean age was 44.0 (SD 8.2) years, 32 PCPs were female (87%), and the years of experience in primary care ranged from 10 to 15 years. General practitioners and practice nurses assessed eligibility in collaboration.

### Study Population

A total of 282 patients with T2DM had reached their treatment targets for HbA_1c_, SBP, and LDL cholesterol at the time of selection. They had a mean age of 63.0 (SD 13.5) years, with a median diabetes duration of 6.6 years (IQR 7.0); 160 patients were male (56.7%) and 184 patients were of Western European origin (184/247, 74.5%; Table 1).

Ineligible patients were older and more often female than eligible patients, they had a higher HbA_1c_ level, a longer diabetes duration, more microvascular and macrovascular complications, and they used insulin more often (Table 1).

**Table 1:** Characteristics of all (N=282), eligible (n=154), and ineligible (n=128) patients for Internet-based self-management.

Characteristics	Patients with well-controlled T2DM^a^, N (%)	Eligible patients, n (%)	Ineligible patients, n (%)	*P* value (eligible vs ineligible)
Total number of patients	282 (100)	154 (54.6)	128 (45.4)	
Age in years, mean (SD)	63.0 (13.5)	59.3 (12.1)	67.6 (13.8)	<.001
Sex, female	122 (43.3)	58 (37.7)	64 (50)	.04
Ethnicity, Western European^b^	184 (74.5)	121 (78.6)	63 (67.7)	.06
Educational level, low^c^	53 (58.2)	39 (52.7)	25 (65.8)	.15
Diabetes duration, years, median (IQR^d^)	6.6 (7.0)	5.5 (5.2)	7.7 (7.5)	.002
HbA_1c_^e^ (mmol/mol), median (IQR^d^)	48.0 (6)	48.0 (7)	49.0 (7)	.01
HbA_1c_ (%),median (IQR^d^)	6.5 (0.6)	6.5 (0.6)	6.6 (0.6)	.01
LDL^f^ cholesterol (mmol/L), median (IQR^d^)	1.90 (0.6)	2.00 (0.7)	1.90 (0.7)	.15
Systolic blood pressure (mm Hg), median (IQR^d^)	128 (15)	128 (18)	129 (17)	.06
BMI^g^ (kg/m^2^), median (IQR^d^)	28.0 (6.1)	27.6 (5.8)	28.7 (6.8)	.33
Microvascular complications	77 (28.6)	33 (22.9)	44 (35.2)	.03
Cardiovascular disease	66 (23.4)	29 (18.8)	37 (28.9)	.05
Lifestyle advice only	49 (17.4)	31 (20.1)	18 (14.1)	.18
Insulin use	29 (10.3)	10 (6.5)	19 (14.8)	.02

^a^T2DM: type 2 diabetes mellitus.

^b^Ethnicity N=247; n=154 in eligible patients and n=93 in ineligible patients.

^c^Education N=112; n=74 in eligible patients and n=38 in ineligible patients.

^d^IQR: interquartile range.

^e^HbA_1c_: hemoglobin A_1c_.

^f^LDL: low-density lipoprotein.

^g^BMI: body mass index.

**Figure 1:**
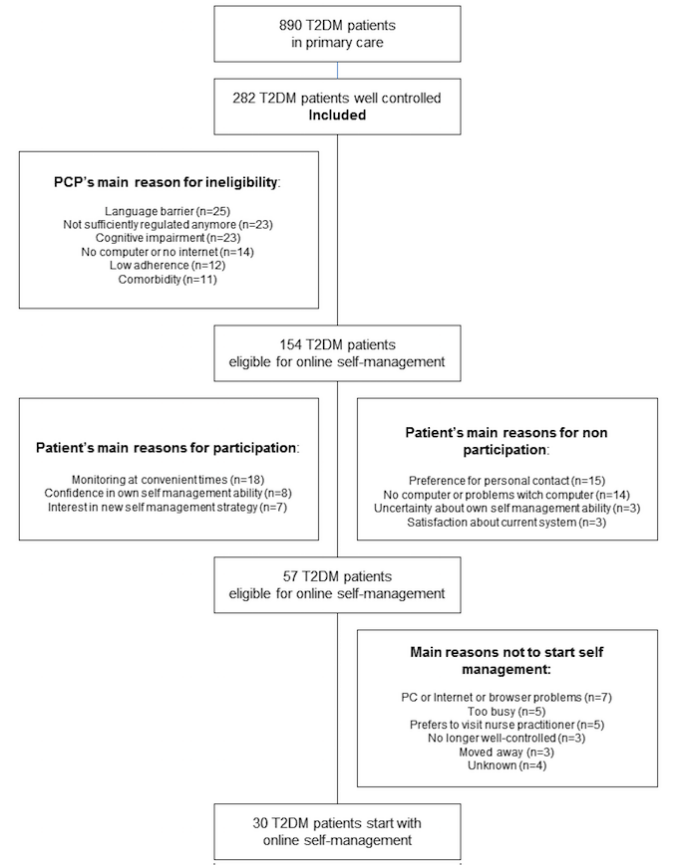
Flow chart of the study.

Slightly more than half of the patients with well-controlled T2DM (154/282, 54.6%) were considered eligible for Internet-based self-management (Figure 1). The remaining patients were considered incapable of using the Internet-based self-management program mainly because of “language barrier” (n=25), “not sufficiently controlled diabetes anymore” (n=23), or “cognitive impairment” (n=23; Figure 1).

Older patients were more likely to be considered ineligible for Internet-based self-management by their PCPs compared with younger patients (OR for each year 1.05, 95% CI 1.03-1.08, *P*<.01). After adding ethnicity, patients with non–Western European ethnicity (OR 3.64, 95% CI 1.67-7.92, *P*<.01) and those with a longer diabetes duration (OR for each year 1.56, 95% CI 1.04-2.34, *P*=.03) were also more likely to be considered ineligible by their PCP (Table 2).

**Table 2:** Ineligibility of patients with well-controlled type 2 diabetes for Internet-based self-management; models are adjusted for health center.

Characteristics	Model 1		Model 2 (including ethnicity)	
	Odds ratio (95% CI)	*P* value	Odds ratio (95% CI)	*P* value
Age in years	1.05 (1.03-1.08)	<.01	1.06 (1.03-1.09)	<.001
Sex, female	1.55 (0.90-2.67)	.11	1.27 (0.68-2.38)	.45
Diabetes duration in years	1.07 (0.78-1.47)	.69	1.56 (1.04-2.34)	.03
Microvascular complications (present)	1.14 (0.62-2.11)	.68	0.94 (0.46-1.94)	.87
Cardiovascular disease (present)	1.19 (0.61-2.31)	.61	1.41 (0.67-3.00)	.37
Insulin use (present)	1.65 (0.63-4.30)	.31	1.63 (0.66-4.79)	.37
Lifestyle advice only (present)	0.61 (0.29-1.28)	.19	0.56 (0.23-1.36)	.20
Body mass index (index scores)	1.04 (0.98-1.11)	.16	1.05 (0.98-1.12)	.15
Ethnicity (non–Western European)	-	-	3.64 (1.67-7.92)	.001

### Willingness to Participate: Preferences of the Patients Themselves

The 154 eligible patients were invited to participate. Their mean age was 59.3 (SD 12.1) years, their median diabetes duration was 5.5 years (IQR 5.2), 62.3% (96/154) of the patients were male, and 78.6% (121/154) were Western European (Table 3). Of the 154 patients, 57 (37%) were willing to participate (Figure 1). Nonparticipants were older, had more often a low educational level, and had a longer diabetes duration than participants (Table 3).

**Table 3:** Characteristics of patients invited for Internet-based self-management.

Characteristics	Total population, N (%)	Participating patients, n (%)	Nonparticipating patients, n (%)	*P* value
Total number of patients	154 (100)	57 (37)	97 (63)	
Age in years, mean (SD)	59.3 (12.1)	55.2 (10)	61.7 (13)	.001
Sex, male	96 (62.3)	40 (70)	56 (58)	.12
Ethnicity (Western European)	121 (78.6)	47 (83)	74 (76)	.37
Educational level, low (n=74)	39 (52.7)	15 (50)	24 (55)	.04
Diabetes duration, years, median (IQR^a^)	5.5 (5.2)	4.7 (6)	5.7 (6)	.05
HbA_1c_^b^ (mmol/mol), median (IQR^a^)	48.0 (7)	47.0 (8)	48.0 (6)	.13
HbA_1c_ (%),median (IQR^a^)	6.5 (0.6)	6.5 (1)	6.5 (1)	.13
LDL^c^ cholesterol (mmol/L), median (IQR^a^)	2.0 (0.7)	2.0 (1)	2.0 (1)	.95
Systolic blood pressure (mm Hg), median (IQR^a^)	128 (18)	122 (18)	128 (15)	.06
BMI^d^ (kg/m^2^), median (IQR^a^)	27.6 (5.8)	27.0 (5)	28.5 (6)	.07
Microvascular complications	33 (22.9)	8 (28)	25 (15)	.09
Cardiovascular disease	29 (18.8)	10 (18)	19 (20)	.75
Lifestyle advice only	31 (20.1)	10 (18)	21 (22)	.54
Oral diabetes medication use	121 (78.6)	47 (83)	74 (76)	.37
Statin use	115 (74.7)	38 (67)	77 (79)	.08
Insulin use	10 (6.5)	4 (7.0)	6 (6)	.84

^a^IQR: interquartile range.

^b^HbA_1c_: hemoglobin A_1c_.

^c^LDL: low-density lipoprotein.

^d^BMI: body mass index.

Treatment preference was motivated by 48.1 % of the eligible patients (74/154). The reason “monitoring at a convenient time” was mentioned most often (18 patients). The reasons mentioned most often for nonparticipation were “preference for personal contact or visiting nurse practitioner” (n=15 patients) and “no computer or problems working with computer” (n=14 patients; Figure 1).

### Questionnaires

Of the 57 patients willing to participate, 45 returned the 3 questionnaires; from the 97 nonparticipants, 21 questionnaires were received. Nonparticipants less often thought that their disease would last their entire life (median 8.0 vs 10.0, *P*=.03) and they were more satisfied with their current treatment than participants. There was no significant difference in quality of life between the 2 groups (Table 4).

**Table 4:** Quality of life, illness perceptions, and treatment satisfaction.

Questionnaires	Participating patients (n=57)	Nonparticipating patients (n=97)	*P* value
Number of questionnaires returned, n (%)	45 (79)	21 (22)	
EQ-5D health state utility score, median (IQR^a^)	1.0 (0.16)	0.89 (0.19)	.20
EQ VAS^b^ (range 0-100), mean (SD)	75.9 (11.5)	77.2 (13.7)	.68
BIPQ^c^ consequences (range 0-10) median (IQR^a^)	3.0 (5)	1.0 (5)	.27
BIPQ timeline (range 0-10), median (IQR^a^)	10.0 (2)	8.0 (4)	.03
BIPQ personal control (range 0-10), median (IQR^a^)	8.0 (1)	6.0 (4)	.21
BIPQ treatment control (range 0-10), median (IQR^a^)	8.0 (2)	8.0 (2)	.06
BIPQ identity(range 0-10), median (IQR^a^)	2.0 (3)	2.0 (4)	.58
BIPQ concern (range 0-10), median (IQR^a^)	4.0 (5)	2.0 (7)	.89
BIPQ understanding (range 0-10), median (IQR^a^)	8.0 (2)	7.0 (3)	.16
BIPQ emotional response (range 0-10), median (IQR^a^)	2.0 (4)	1.0 (3)	.33
DTSQ^d^ total score (range 0-48), median (IQR^a^)	40.0 (6)	44.0 (10)	.05

^a^IQR: interquartile range.

^b^EQ VAS: EQ visual analogue scale.

^c^BIPQ: Brief Illness Perception Questionnaire.

^d^DTSQ: Diabetes Treatment Satisfaction Questionnaire.

Multivariate analysis showed that older patients were more likely to not participate compared with younger patients (OR for each year 1.06, 95% CI 1.02-1.10, *P*<.01). The same held true for non–Western European ethnicity (OR 3.33, 95% CI 1.25-8.88, *P*=.02) and for those with a higher BMI (OR for each kg/m^2^ 1.11, 95% CI 1.02-1.22, *P*=.02).

Finally, only 30 patients started the Internet-based self-management support program (Figure 1). Predominantly, practical issues (such as problems related to Internet access) hindered implementation of the Internet-based self-management program. Other patients still preferred to visit the nurse practitioner.

### Active Participation in the First Year of the Internet-based Self-Management Program

Patients started their first Internet-based support session 3 months after providing informed consent. Depending on their starting date, patients in the Internet-based self-management support program had completed one or more sessions by the date of the database lock. None of the patients stopped the Internet-based self-management monitoring sessions in the first year after implementation. Mean values of fasting blood glucose, SBP, and BMI remained stable during this study period.

Most patients used oral diabetes medication (80%). Antihypertensive medication was used by half of the patients and 67% (38/57) of the patients used a statin. In 4 cases, medication was changed in response to the Internet-based contact, twice during the first and twice during the second Internet-based contact. Furthermore, the following personalized advice was given:

1. In 83% of the self-management monitoring sessions the patient received a message that their entered data were within target.

2. In the 64 self-management monitoring sessions performed, 11 patients were advised to contact their PCP the next working day, either because of a very high self-reported blood pressure value (n=4) or because of reported diabetes-related health problems in the previous 3 months (n=7);

3. Two patients received a message to contact their PCP the same day, 1 patient because of an entered low blood pressure value and 1 patient because of diabetes-related health problems; both patients followed the advice.

## Discussion

### Principal Findings

This study explored the reach of an Internet-based self-management support program including a 75% decrease in personal contact, replaced by Internet-based personalized advice, in T2DM patients with good cardiometabolic control. Results showed that the PCPs perceived almost half of their own patients with well-controlled T2DM as ineligible for this approach, mainly because of cognitive impairment and language barriers. Of the 154 eligible patients, 37% (57/154) chose to participate. The main reason to participate was better time management, whereas the reasons mentioned most often for nonparticipation were a preference to visit the nurse practitioner and not having a computer. Older patients, patients with non–Western European ethnicity, and patients with a high BMI were less likely to participate. Of the 57 patients who chose to participate, only 30 patients started the Internet-based program, mostly because Internet and browser problems. Mean cardiometabolic values remained stable during participation in the Internet-based self-management support program.

Internet-based self-management support as a replacement for part of the regular care might facilitate patient centeredness and time-effectiveness. Patients can manage their disease—with personalized goals—and can perform the monitoring at home at a convenient time; they do not have to visit the health center and are not absent from work, sport, or family. Moreover, it enables PCPs to give more attention to patients with poorly controlled T2DM. However, the applicability of Internet-based self-management support showed to be limited, even in this group of people with well-controlled T2DM.

Participants of the Internet-based program were younger than nonparticipants, suggesting that older patients are less comfortable working with Internet-based self-management with their personal computer. This suggestion is supported by the main reason for nonparticipation as mentioned by the eligible patients. Previous research showed that elderly patients often have poor technical skills in this respect [25].

Our finding that T2DM patients with higher education were more willing to take over some monitoring duties is in concordance with previous research [26]. Patients who were less satisfied with their current treatment might have been more willing to participate in Internet-based self-management because they prefer a more active role in their own treatment.

Patient of Western European origin, in first instance based on the PCPs’ selection but also based on the patients’ own preference were more often among the participants than those with Non-Western European origin. Self-care behaviors differ by ethnicity and self-efficacy. Differences in perceptions have an impact on self-care behavior [27-29]. The reason that patients with non–Western European ethnicity seemed less motivated to participate might be due to ethnic differences in illness perceptions [30]. Non–Western European patients might perceive their diabetes more often as a harmless condition that could be cured, compared with Western European patients [31]. These findings suggest that culturally tailored messages about diabetes self-care might be needed. However, other reasons for nonparticipation should also be considered, such as health literacy.

A strong aspect of this study is the real-life setting in which the true reach of an Internet-based self-management support program with all the related difficulties of its implementation could be demonstrated. However, against that background this study had some limitations. First, patients were considered to have good cardiometabolic control based on data retrieved from the patient records in August 2014, whereas the patients were enrolled in 2015. The time interval between the “eligibility check” by the PCPs and the invitation resulted in a number of patients who could not participate anymore.

Second, one could state that the reach of the program was limited because patients with well-controlled T2DM were offered the opportunity to participate in the Internet-based self-management program only when their PCP judged them as eligible. Therefore, not all patients with well-controlled T2DM were asked to participate. This strategy was chosen for patient safety. However, the indications for selection by the PCPs, and afterward the patients’ preferences, were rather similar: younger age, Western European ethnicity, and shorter diabetes duration. A shared decision-making process is a realistic and more elegant option to determine the eligibility of patients to participate in an Internet-based self-management program and to evaluate the preferences of all patients with well-controlled T2DM.

Third, the number of patients who actually started the Internet-based self-management support program was low. There were some Internet and browser problems with the implementation of the Internet-based self-management support program, leading to fewer participants to start the program. However, this represents implementation in a real-life setting.

### Conclusions

This study showed that PCPs consider about half of all patients with well-controlled T2DM eligible for an Internet-based self-management support program and that about 1 out of 3 eligible patients is willing to participate. Almost half of the patients who chose to participate did not actually start the program, demonstrating that implementation of such a program is difficult and its applicability is limited. Although only 10% of all patients with well-controlled T2DM eventually started the program, this number is relevant, given the huge numbers of people with T2DM. For example, with about 800,000 people with T2DM in the Netherlands and 25%-30% with good control, the use of our Internet-based self-management support program could hypothetically result in a reduction of nearly 70,000 practice visits a year, which might diminish the diabetes burden on the health care system.

## Abbreviations

BIPQ: Brief Illness Perception Questionnaire
BMI: body mass index
DSME: diabetes self-management education
DSME/S: diabetes self-management education and support
DTSQ: Diabetes Treatment Satisfaction Questionnaire
eGFR: estimated glomerular filtration rate
EQ VAS: EQ visual analogue scale
HbA1c: hemoglobin A1c
IQR: interquartile range
LDL: low-density lipoprotein
OR: odds ratio
PCP: primary care provider
SBP: systolic blood pressure
SCORE: Systematic Coronary Risk Evaluation
T2DM: type 2 diabetes mellitus
